# Temporal profiling of Kv1.3 channel expression in brain mononuclear phagocytes following ischemic stroke

**DOI:** 10.1186/s12974-019-1510-8

**Published:** 2019-06-01

**Authors:** Tianwen Gao, Syed Ali Raza, Supriya Ramesha, Ngozi V. Nwabueze, Amelia J. Tomkins, Lihong Cheng, Hailian Xiao, Manuel Yepes, Srikant Rangaraju

**Affiliations:** 1 Department of Neurology, Emory University, Atlanta, GA USA; 2 Division of Neuropharmacology and Neurologic Diseases, Yerkes National Primate Research Center, Atlanta, GA USA; 3 Department of Neurology, Veterans Affairs Medical Center, Atlanta, GA USA; 4 Xiangya Hospital, Central South University, Changsha, Hunan 410008 China

**Keywords:** Ischemic stroke, Kv1.3, Macrophage, Microglia, Middle cerebral artery occlusion, Neuroinflammation

## Abstract

**Background:**

Microglia and CNS-infiltrating monocytes/macrophages (CNS-MPs) perform pro-inflammatory and protective anti-inflammatory functions following ischemic stroke. Selective inhibition of pro-inflammatory responses can be achieved by Kv1.3 channel blockade, resulting in a lower infarct size in the transient middle cerebral artery occlusion (tMCAO) model. Whether beneficial effects of Kv1.3 blockers are mediated by targeting microglia or CNS-infiltrating monocytes/macrophages remains unclear.

**Methods:**

In the 30-min tMCAO mouse model, we profiled functional cell-surface Kv1.3 channels and phagocytic properties of acutely isolated CNS-MPs at various timepoints post-reperfusion. Kv1.3 channels were flow cytometrically detected using fluorescein-conjugated Kv1.3-binding peptide ShK-F6CA as well as by immunohistochemistry. Quantitative reverse-transcriptase polymerase chain reaction (qRT-PCR) was performed to measure Kv1.3 (Kcna3) and Kir2.1 (Kcnj2) gene expression. Phagocytosis of 1-μm microspheres by acutely isolated CNS-MPs was measured by flow cytometry.

**Results:**

In flow cytometric assays, Kv1.3 channel expression by CD11b^+^ CNS-MPs was increased between 24 and 72 h post-tMCAO and decreased by 7 days post-tMCAO. Increased Kv1.3 expression was restricted to CD11b^+^CD45^low^Ly6c^low^ (microglia) and CD11b^+^CD45^high^Ly6C^low^ CNS-MPs but not CD11b^+^CD45^high^Ly6c^high^ inflammatory monocytes/macrophages. In immunohistochemical studies, Kv1.3 protein expression was increased in Iba1^+^ microglia at 24-48 h post-tMCAO. No change in Kv1.3 mRNA in CNS-MPs was observed following tMCAO.

**Conclusions:**

We conclude that resident microglia and a subset of CD45^high^Ly6c^low^ CNS-MPs are the likely cellular targets of Kv1.3 blockers and the delayed phase of neuroinflammation is the optimal therapeutic window for Kv1.3 blockade in ischemic stroke.

**Electronic supplementary material:**

The online version of this article (10.1186/s12974-019-1510-8) contains supplementary material, which is available to authorized users.

## Introduction

Ischemic stroke is a leading cause of mortality and disability, and there is an urgent need for therapies that can limit ischemic brain injury beyond the acute phase following ischemic stroke [1–4]. Neuroinflammation mediated by innate immune cells of the CNS (microglia and CNS-infiltrating macrophages collectively called CNS mononuclear phagocytes or CNS-MPs) comprises of complex pro-inflammatory responses that promote neuronal injury as well as anti-inflammatory/neuroprotective responses [3, 5]. Potentially protective anti-inflammatory CNS-MP phenotypes predominate in the first 1–2 days following ischemic injury whereas detrimental pro-inflammatory phenotypes increase after 48 h [6–8]. Identifying the key regulators of detrimental and protective CNS-MP responses can facilitate discovery of novel targets for ischemic stroke. The recently proven efficacy of endovascular thrombectomy for acute ischemic stroke has created exciting opportunities to translate neuro-immunomodulatory and neuroprotective strategies that target disease mechanisms beyond the acute phase [9, 10].

The diverse transcriptional profiles adopted by CNS-MPs have been recently revealed by transcriptomic studies which found an emergence of unique disease-associated microglial (DAM) profiles and downregulation of homeostatic genes in mouse models of chronic neuroinflammation, aging, and neurodegeneration [11–13]. More recently, we identified molecular heterogeneity within DAM which is comprised of independently regulated pro-inflammatory and anti-inflammatory DAM sub-profiles [13]. The potassium channel Kv1.3 was identified as a marker and regulator of pro-inflammatory DAM [13], and inhibition of Kv1.3 channels was found to be beneficial in Alzheimer’s disease models as well as in the transient middle cerebral artery occlusion (tMCAO) ischemic stroke model [13–15]. Although increased expression of Kv1.3 channels have been confirmed in acutely isolated CNS-MPs by electrophysiology, it is unclear whether microglia and/or CNS-infiltrating macrophages are the primary cell type with increased Kv1.3 channel expression [14].

We have applied a novel flow cytometric assay of functional cell-surface Kv1.3 channels coupled with parallel phagocytic profiling of acutely isolated CNS-MPs [16] at early and delayed timepoints following tMCAO, to define the temporal profiles of Kv1.3 channel expression within CNS-MP subsets.

## Materials and methods

### Reagents

Fluorescein-conjugated ShK-F6CA was purchased from Peptides International (Louisville, KY) [17, 18]. Fluorophore-conjugated antibodies for flow cytometry were obtained from BD Biosciences (CD11b-APC-Cy7, CD45-PE-Cy7, CD45-FITC, Ly6c-PE) [13]. Polystyrene phycoerythrin-fluorescent 1-μm microspheres (Thermo Fisher #F13083) were used for phagocytosis assays [16]. Percoll was purchased from Sigma-Aldrich (#P1644).

### Animals

Male C57BL/6 J mice (JAX 000664) aged 8–12 weeks were housed in the Department of Animal Resources at Emory University under standard conditions. Institutional Animal Care and Use Committee approval was obtained, and all in vivo studies were performed in strict accordance with the Guide for the Care and Use of Laboratory Animals of the National Institute of Health, and in compliance with the ARRIVE guidelines.

### Transient middle cerebral artery occlusion (tMCAO) model

After anesthesia (isoflurane) and exposure of the carotid artery, a 6-0 silk suture was advanced from the external carotid artery into the internal carotid artery until the origin of the middle cerebral artery on the ipsilateral side [19, 20]. The suture was then withdrawn after 30 min of cerebral ischemia. Laser Doppler flow was used to monitor cerebral perfusion at baseline, at time of MCAO, immediately after recanalization, and 5 min post-recanalization. Animals with > 75% decreased middle cerebral blood flow and > 75% reperfusion were included. In total, 32 mice were included for flow cytometric analyses (30 min: *n =* 8, 24 h: *n =* 7, 48 h: *n =* 8, 72 h: *n =* 4, 7 days: *n =* 5). Sham surgery controls were performed (*n =* 3 per condition). Transcranial Doppler flow data demonstrating effective MCAO and complete reperfusion for mice included in the study are shown in Additional file 1: Figure S1.

### CNS-MP isolation

After tMCAO, mice were euthanized at 30 min, 24 h, 48 h, 72 h, and 7 days followed by cardiac perfusion. CNS-MPs were acutely isolated from ipsilateral and contralateral hemispheres using Percoll density (70%/35%) centrifugation as previously described [16, 18]. After myelin removal, mononuclear cells were re-suspended in saline or Trizol. From each hemisphere, half was used for RNA extraction and the other half was used for flow cytometry.

### Flow cytometry

Functional cell-surface Kv1.3 channel expression in acutely isolated CNS-MPs was measured using a validated fluorescein-conjugated ShK-F6CA assay [13, 18, 21]. Cells were incubated with 10 nM ShK-F6CA along with fluorophore-conjugated CD11b (APC-Cy7), CD45 (PE-Cy7), and Ly6c (PE) antibodies for 30 min and then washed twice with cold PBS to remove any unbound antibodies. Compensation controls were run along with appropriate negative/isotype controls [18]. Live mononuclear cells were first gated based on forward and side scatter profiles, and then single cells were gated and then further gated for CD11b^+^ CNS-MPs. CD11b^+^ CNS-MPs were gated into CD45^low^Ly6c^low^, CD45^high^Ly6c^low^, and CD45^high^Ly6c^high^ subpopulations. Each CNS-MP subpopulation [22, 23] was evaluated for ShK-F6CA labeling.

### Immunohistochemical studies

Following 30 min MCAO, mice were euthanized at 1 h, 24 h, and 48 h timepoints and brains were post-fixed in 4% PFA for 24 h, then transferred to 30% sucrose for another 24 h, and then sectioned (30 μm) and preserved in cryoprotectant solution. Free-floating sections were blocked with 10% horse serum for 1 h, then incubated with primary antibodies (anti-Kv1.3 mAb 1:100 UC Davis/NIH NeuroMab clone L23/27, anti-Tmem119 rabbit mAb 1:100 #ab209064, or anti- Iba1 rabbit mAb 1:300 #ab178846) at 4 °C overnight. Sections were then washed and incubated with fluorophore-conjugated secondary antibodies (1:500) for 30 min and then mounted on slides and dried. Hard-mounting medium (with DAPI-VectorLabs # H1500) was used to mount sections which were then imaged on an immunofluorescence microscope (Microscope: Olympus BX51 and camera: Olympus DP70) at 20× and 60× (oil immersion) magnifications. The ipsilateral (stroke) hemisphere was marked prior to sectioning. At least five fields in the peri-infarct region ipsilaterally and corresponding region on contralateral hemisphere were imaged per mouse (*n =* 2–3 mice per timepoint). All immunofluorescence images were processed using ImageJ software (version 1.52a).

### Phagocytosis assay

Acutely isolated CNS-MPs were incubated with phycoerythrin (PE)-conjugated microspheres (1:100) at 37 °C for 30 min, washed, and labeled with fluorophore-conjugated anti-CD11b (APC-Cy7) and anti-CD45 (FITC) antibodies. Microsphere phagocytosis in CNS-MPs was assessed as the proportion of cells taking up > 1 microsphere as previously reported [16].

### Quantitative reverse-transcriptase polymerase chain reaction (qRT-PCR)

CNS-MP RNA extracted in Trizol was purified for qRT-PCR [18, 24]. RNA was reverse-transcribed to cDNA (Ambion), and qRT-PCR was performed (7500 Fast RT-PCR instrument, Applied Biosystems) using cDNA, TaqMan PCR Master Mix and gene-specific TaqMan probes (Applied Biosystems) against Kcna3 (Mm00434599_s1), Kcnj2 (m00434616_m1), Ptgs2 (Mm00478374_m1), and Hprt (Mm03024075_m1) in duplicate. Relative gene expression was normalized to Hprt and calculated using the 2ΔΔC_T_ method [25].

### Statistical considerations

GraphPad Prism version 7.0 and SPSS Version 24 were used for statistical analyses. Pairwise comparisons were performed using *T*-tests (independent sample, two-tailed, unequal variance). Non-parametric Mann-Whitney *U* test was used for pairwise comparisons of flow cytometric data. The statistical significance level was set at *p* < 0.05.

## Results

### CNS infiltration by peripherally derived monocytes/macrophages at 48–72 h post-tMCAO

**Fig. 1:**
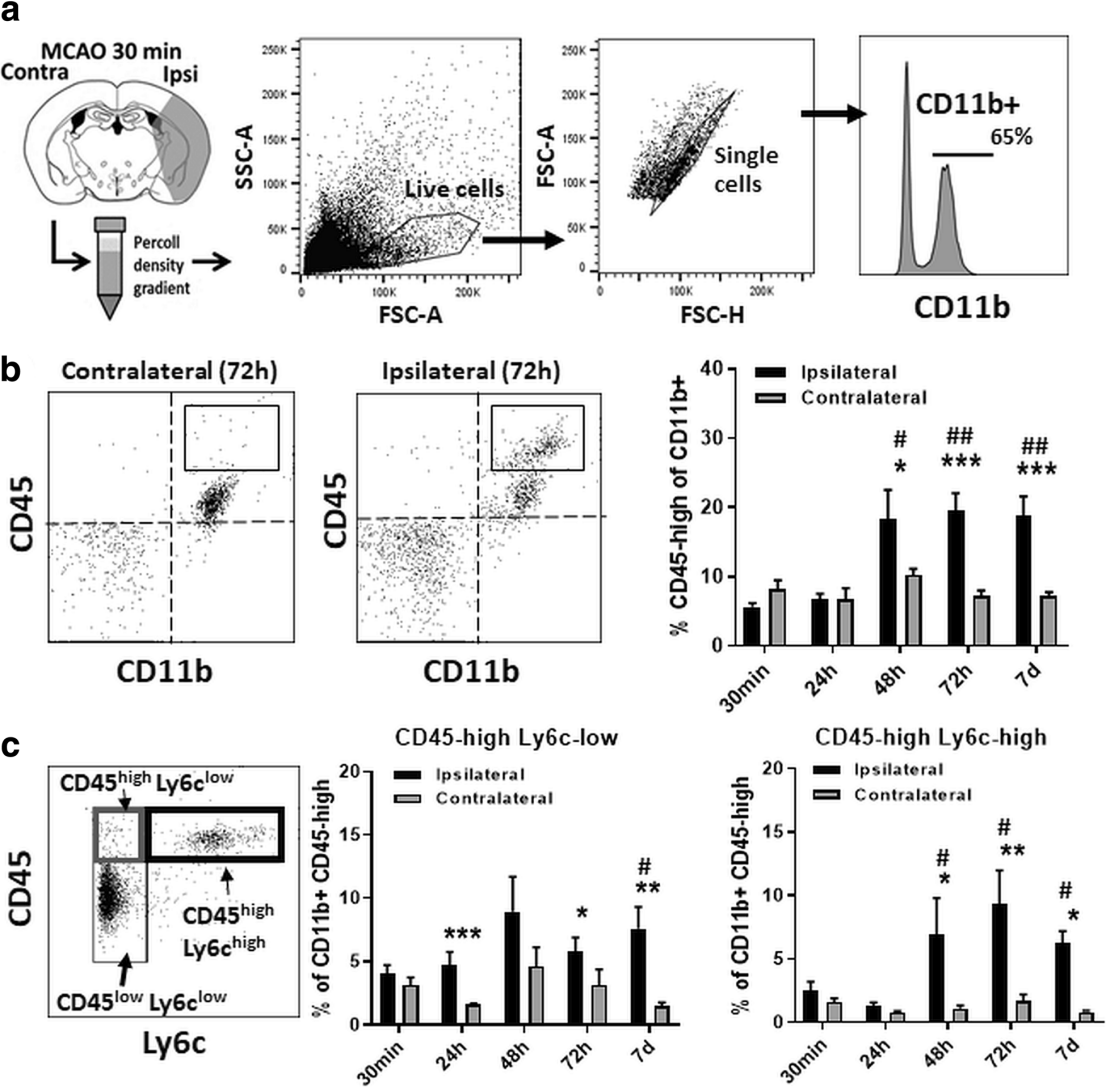
Flow cytometric characterization of populations of CNS immune cells in the tMCAO model. **a** Experimental design and flow cytometric gating strategy: Following tMCAO, CNS-MPs were acutely isolated at 30 min, 24 h, 48 h, 72 h, and 7 days timepoints and characterized by flow-cytometry based on CD11b, CD45, and Ly6c expression. In independent experiments with viability indicators, viability of > 99% was confirmed using this gating strategy. **b** Comparison of CD45^high^ cells within CD11b^+^ CNS-MPs. Quantitative analysis is shown on the right. **c** Subpopulations of CD11b^+^ CNS-MPs based on CD45 and Ly6c expression and temporal patterns post-tMCAO. An example of CD11b^+^ subpopulations from the ischemic hemisphere at 72 h post-tMCAO is shown on the left. Quantitative analysis of relative proportions of Ly6c^high^ and Ly6c^low^ subsets of CD11b^+^CD45^high^ cells in ipsilateral and contralateral hemispheres at various timepoints is shown. *N =* 4–8 mice/group, error bars represent standard error of mean. *indicates comparisons between hemispheres (*p < 0.05, **p < 0.01, ***p < 0.005). ^#^indicates comparisons within ipsilateral hemispheres across timepoints using 30 min timepoint as reference (^#^*p <* 0.05, ^##^*p <* 0.01, ^###^*p <* 0.005)

Effective MCAO (18% baseline) and effective reperfusion (108% of baseline) were achieved in our 30-min tMCAO model (Additional file 1: Figure S1a). At 24 h, 2,3,5-triphenyltetrazolium chloride (TTC) staining demonstrated cerebral infarction as expected in the ipsilateral hemisphere (Additional file 1: Figure S1b). We characterized CD11b^+^ CNS-MPs in the ipsilateral and contralateral hemispheres (excluding the cerebellum) following tMCAO at 30 min to 7 days timepoints (Fig. 1a). As compared to 30 min following tMCAO, we observed a significant increase in the proportions of CD11b^+^CD45^high^ CNS-MPs at 48 h (*p* = 0.02), 72 h (*p* = 0.00014), and 7 days (*p* < 0.00001) following tMCAO in the ipsilateral hemisphere but not in the contralateral hemisphere (Fig. 1b and Additional file 2: Figure S2a). Among all CD11b^+^ CNS-MPs, we also observed a significant increase in the proportions of CD45^high^Ly6c^high^ CNS-infiltrating macrophages/inflammatory monocytes at 48 h (*p =* 0.023) and 72 h (*p =* 0.01) following tMCAO, and in proportions of CD11b^+^CD45^high^Ly6c^low^ non-inflammatory monocytes/activated microglia at 48 h to 7 days (*p =* 0.04) ipsilaterally following tMCAO [26, 27] (Fig. 1c). As compared to CD45^high^Ly6c^low^ CNS-MPs, the increase in proportions of CD45^high^Ly6c^high^ CNS-MPs post-tMCAO was more robust (Fig. 1c). No changes in CNS-MP subset proportions were observed on the contralateral side. There was also an early increase in CNS CD11b^neg^CD45^high^ lymphocytes ipsilaterally at 30 min which rapidly decreased by 24 h (Additional file 2: Figure S2b). These data confirm that a wave of CNS infiltration by inflammatory monocytes occurs between 48 h to 7 days post-tMCAO [26, 27].

### Temporal flow cytometric profiling of Kv1.3 channels in CNS-MPs following tMCAO

**Fig. 2:**
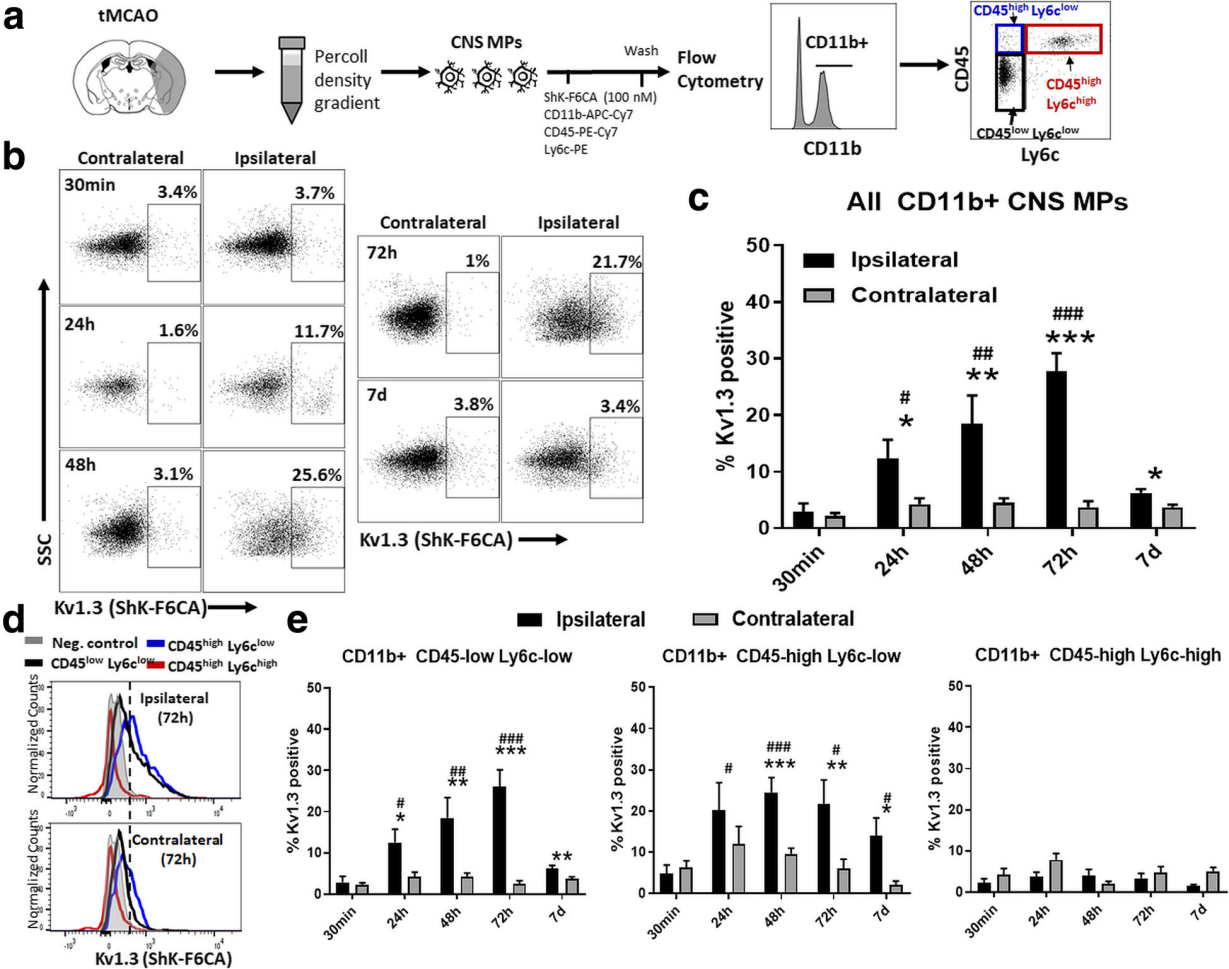
Temporal profiling of functional cell-surface Kv1.3 channels in CNS-MPs following tMCAO. **a** CNS-MPs following tMCAO were incubated with 100 nM ShK-F6CA along with flow cytometric antibodies. **b** Kv1.3 channel expression by CD11b^+^ CNS-MPs (one example from each timepoint) post-tMCAO. Negative control labeled with non-specific FITC-conjugated IgG. **c** Quantitative analysis of Kv1.3 channel expression by CD11b^+^ CNS-MPs at different timepoints following tMCAO. **d** Representative flow cytometric histograms showing distinct Kv1.3 surface expression in CD11b^+^CD45^low^ and both Ly6^low^ and Ly6c^high^ subsets of CD11b^+^CD45^high^ cells. **e** Quantitative comparison of Kv1.3 channel expression by distinct CNS-MP subsets following tMCAO. *N =* 4–8 mice/group. Error bars represent standard error of mean. *indicates comparisons between hemispheres (* *p <* 0.05, **p < 0.01, ***p < 0.005). ^#^indicates comparisons within ipsilateral hemispheres across timepoints using 30 min timepoint as reference (^#^*p <* 0.05, ^##^*p <* 0.01, ^###^*p <* 0.005)

We applied a flow cytometric assay of functional cell-surface Kv1.3 channels (ShK-F6CA) to describe the temporal profiles of Kv1.3 expression following tMCAO and identify the CNS-MP subsets with highest Kv1.3 channel expression (Fig. 2a) [13, 18, 21]. ShK-F6CA is a selective and highly potent peptide blocker of the Kv1.3 channel pore which requires the tetramerization of four Kv1.3 monomers. Unlike most traditional flow cytometric probes which recognize epitopes on proteins, ShK-F6CA binds only to functional Kv1.3 channels that are expressed on the cell surface and we and others have validated this approach to accurately measure Kv1.3 expression in acutely isolated CNS-MPs [13, 18, 21, 28]. We observed a robust and gradual ipsilateral increase in ShK-F6CA fluorescence labeling of Kv1.3 channels in CD11b^+^ CNS-MPs at 24 h (*p =* 0.017), 48 h (*p =* 0.009), and 72 h (*p <* 0.0001) post-tMCAO that nearly resolved by 7 days post-tMCAO (Fig. 2b, c). Minimal ShK-F6CA fluorescence was observed in CD11b^+^ CNS-MPs from the contralateral hemisphere (Fig. 2b, c). Among CD11b^+^ CNS-MP subsets (gating shown in Fig. 2a), we observed that Kv1.3 channels were increased ipsilaterally in CD11b^+^CD45^low^Ly6c^low^ resident microglia (24 h *p =* 0.013, 48 h *p =* 0.009, 72 h *p <* 0.0001) and in CD11b^+^CD45^high^Ly6c^low^ CNS-MPs (24 h *p =* 0.049, 48 h *p =* 0.0005, 72 h *p =* 0.012, 7 days *p =* 0.05) (Fig. 2d, e). However, no significant changes in ipsilateral Kv1.3 expression were observed in CD11b^+^CD45^high^Ly6c^high^ CNS-infiltrating macrophages/inflammatory monocytes following tMCAO (Fig. 2d, e) [26]. ShK-F6CA fluorescence in CD11b^+^CD45^high^ splenic monocytes/macrophages was negligible following sham surgery or post-tMCAO (Additional file 3: Figure S3) suggesting that these subtypes express minimal functional Kv1.3 channels.

### Immunohistochemical validation of increased Kv1.3 protein expression following tMCAO

**Fig. 3:**
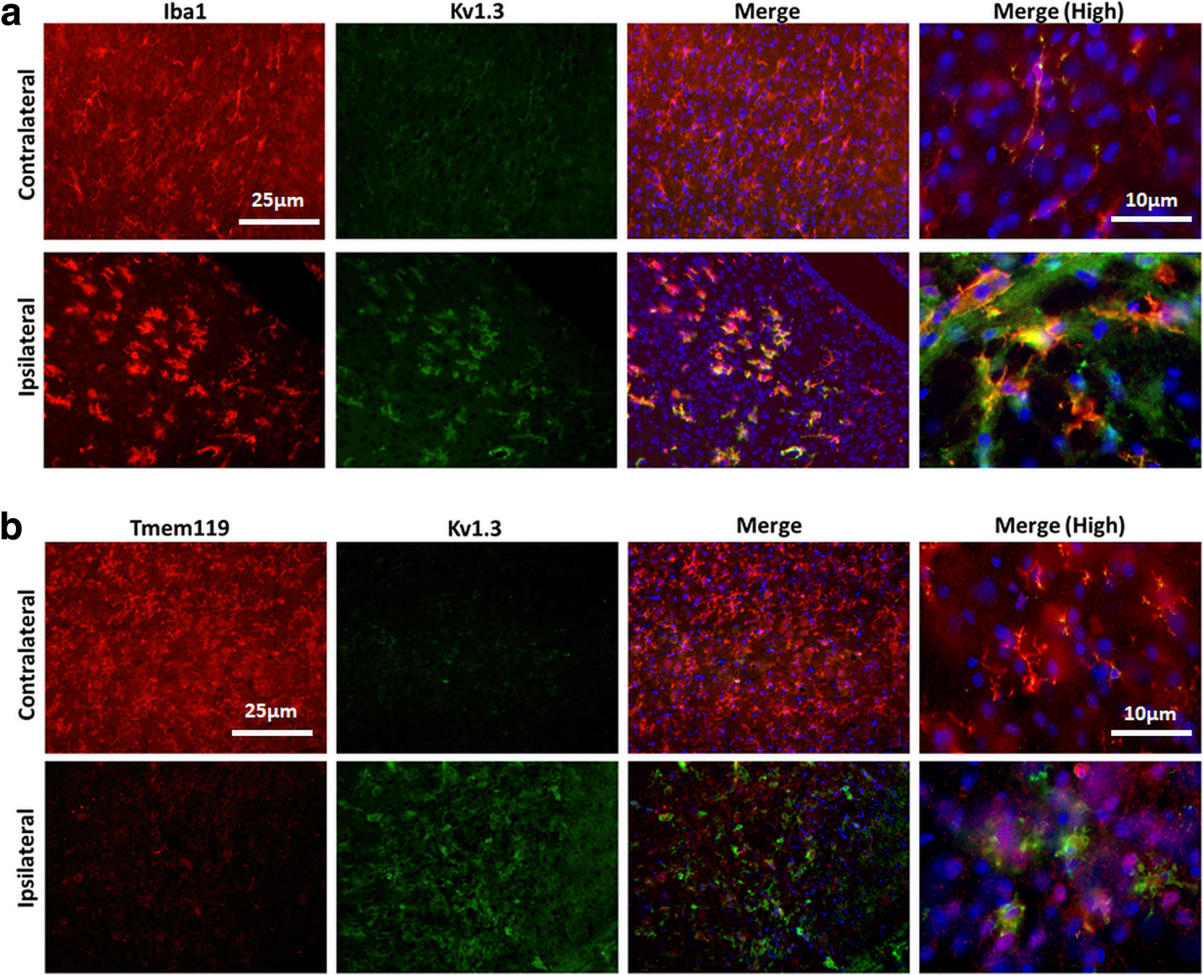
Immunohistochemical validation of increased Kv1.3 protein expression in microglia following tMCAO. **a** Immunofluorescence micrographs from tMCAO brain sections (48 h post-tMCAO) showing microglia/macrophage marker Iba1 (red) as well as Kv1.3 channel expression (green). Minimal Kv1.3 immunoreactivity was noted in the contralateral hemisphere while Kv1.3 positive Iba1^+^ microglia were seen in the peri-infarct region in the ipsilateral hemisphere. Co-localization between Iba1 and Kv1.3 is shown in yellow in the merged image. High-power (60×) images are shown on the right. Red—Iba1; green—Kv1.3; blue—DAPI. Also see Additional file 4: Figure S4 for related imaging from other timepoints. **b** Immunofluorescence micrographs from tMCAO brain sections (48 h post-tMCAO) showing abundant Tmem119 expression by ramified microglia in contralateral hemisphere while ipsilateral Tmem119 expression is significantly reduced. Decreased Tmem119 expression was associated with increased Kv1.3 expression. Co-localization between Iba1 and Kv1.3 is shown in yellow in the merged image. High-power (60×) images are shown on the right. Red—Tmem119; green—Kv1.3; blue—DAPI

To validate our flow cytometric findings, we performed immunofluorescence imaging to characterize Kv1.3 channel protein expression in the brain at 1 h, 24 h, and 48 h following tMCAO (Fig. 3 and Additional file 4: Figure S4). We observed a change in microglial morphology from a ramified state to a more rounded, ameboid, and activated morphology in the ipsilateral hemisphere following tMCAO at 24 and 48 h timepoints (Fig. 3a). At 1 h post-tMCAO, minimal Kv1.3 immunoreactivity was noted in both ipsilateral and contralateral hemispheres and very low-level Kv1.3 positivity was observed in microglia (Additional file 4: Figure S4). At 24 h and 48 h post-tMCAO, we observed increased Kv1.3 immunoreactivity in Iba1^+^ CNS-MPs in the peri-infarct regions (Fig. 3a). We also performed immunofluorescence imaging to measure Kv1.3 expression and Tmem119 post-tMCAO to confirm microglial specificity of Kv1.3 expression (Fig. 3b). At 1 h post-MCAO, Tmem119 immunoreactive microglia exhibited an expected ramified morphology and low levels of Kv1.3 protein expression. At 48 h post-tMCAO, we observed a dramatic decrease in Tmem119 immunoreactivity indicating downregulation of Tmem119 (Fig. 3b). These findings are consistent with downregulation of homeostatic genes/proteins such as Tmem119 [11, 13] following ischemic injury. Based on our flow cytometric and immunohistochemical results as well as the recently published efficacy of selective Kv1.3 channel blockers in the MCAO mouse model [14], the pattern of functional Kv1.3 channel expression following tMCAO indicates that microglia that highly express Kv1.3 channels at 24–72 h post-tMCAO, but not infiltrating monocytes/macrophages, are likely to be the targets of Kv1.3-blocking therapies in stroke.

### Transcriptional alterations in potassium channel genes following tMCAO

**Fig. 4:**
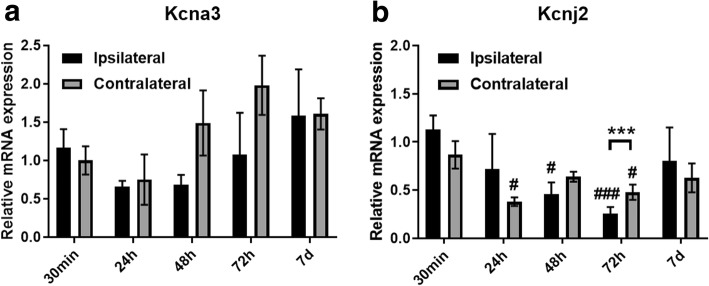
Kv1.3 and Kir2.1 potassium channel gene expression in CNS-MPs post-tMCAO. Comparison of Kv1.3 (**a**) and Kir2.1 (**b**) gene expression in CNS-MPs isolated from ipsilateral and contralateral hemispheres at different timepoints following tMCAO by qRT-PCR (Hprt used as housekeeping gene). *N =* 3 mice/group, error bars represent standard error of mean. *indicates comparisons between hemispheres (*p < 0.05, **p < 0.01, ***p < 0.005) at a particular timepoint. ^#^indicates comparisons within ipsilateral or contralateral hemispheres across timepoints using 30 min timepoint as reference (^#^*p <* 0.05, ^##^*p <* 0.01, ^###^*p <* 0.005)

Since we observed significant alterations in functional Kv1.3 channel expression on the surface as well as at the protein level in microglia at 24–72 h following tMCAO, we determined whether changes in Kv1.3/Kcna3 gene expression in CNS-MPs can account for this increased functional channel expression. In qRT-PCR studies performed on acutely isolated CNS-MPs (≈ 60% CD11b^+^ CNS-MPs [28]) from ipsilateral and contralateral hemispheres following tMCAO, we observed no significant changes in Kcna3 (Kv1.3) mRNA expression in ipsilateral or contralateral hemispheres (Fig. 4a). Interestingly, we observed a significant downregulation in gene expression of an inward-rectifying K channel (Kir2.1/Kcnj2) in CNS-MPs from both ipsilateral (48 h *p =* 0.02, 72 h *p =* 0.004) and contralateral hemispheres (24 h *p =* 0.025, 72 h *p =* 0.03) post-tMCAO (Fig. 4b) [29]. Downregulation of Kir2.1/Kcnj2 was most pronounced in the ipsilateral hemisphere at 72 h post-tMCAO (*p =* 0.004, Fig. 4b). It has been previously reported that increased Kv1.3 channel expression in microglia that is induced by pro-inflammatory stimuli is associated with a reciprocal decrease in Kir2.1 channels while anti-inflammatory stimuli increase Kir2.1 currents [29]. Furthermore, Kv1.3 channels may represent regulators of pro-inflammatory microglial responses while Kir2.1 may regulate anti-inflammatory and protective responses [13, 28]. Based on the minimal changes in Kv1.3/Kcna3 mRNA levels in CNS-MPs following tMCAO, the observed robust changes in functional Kv1.3 channels in microglia post-tMCAO may indicate post-transcriptional regulation of Kv1.3 channels in microglia.

### Characterization of phagocytic properties of CNS-MP subsets following tMCAO

**Fig. 5:**
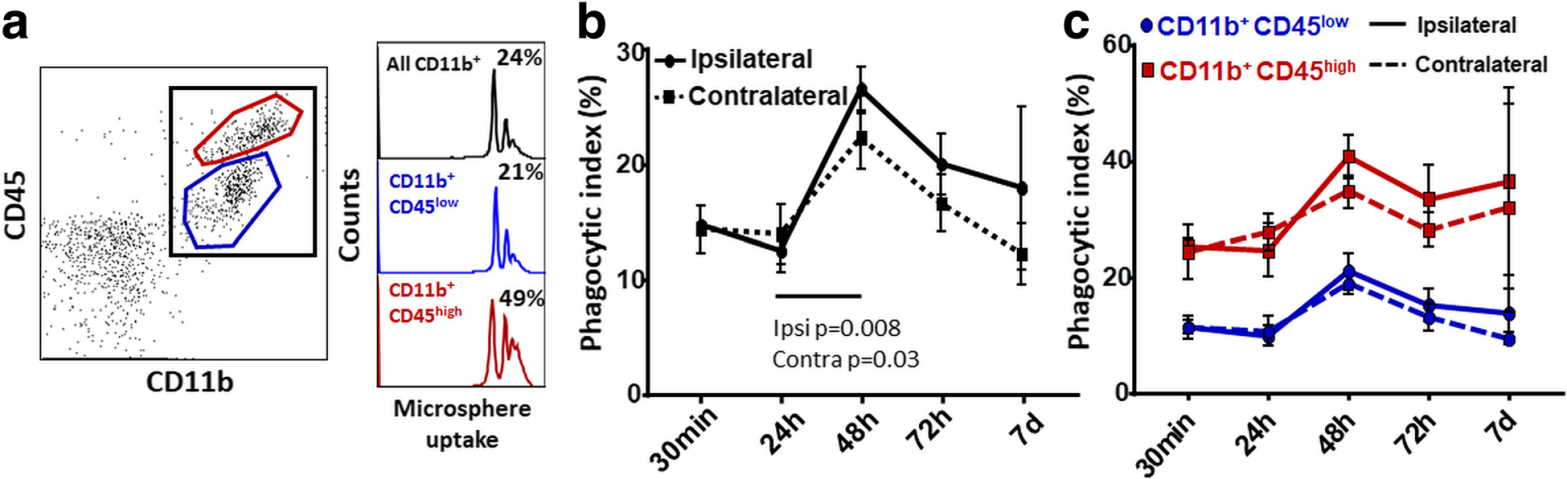
Alterations in phagocytic capacity of CNS myeloid subpopulations following tMCAO. **a** Acutely isolated CNS-MPs were incubated with PE-conjugated microspheres and labeled with CD11b-APC-Cy7 and CD45-FITC antibodies. Uptake of > 1 microsphere (phagocytic index) was measured by flow cytometry. Typical phagocytic indices for all CD11b^+^ CNS myeloid cells as well as CD45^low^ and CD45^high^ subpopulations at 48 h post-tMCAO (ipsilateral hemisphere) are shown (right). **b**, **c** Results from comparison of phagocytic capacity of all CD11b^+^ CNS-MPs as well as CD45^low^ and CD45^high^ subpopulations in ipsilateral and contralateral hemispheres following tMCAO. For all analyses, *n =* 3–4 mice/group, error bars represent standard error of mean, *p < 0.05, **p < 0.01

Microglia and CNS-infiltrating macrophages are involved in phagocytic clearance following CNS injury [3, 30]. We recently showed that higher phagocytic activity is observed in CNS-MPs with higher Kv1.3 channel expression and that phagocytic properties can be augmented by Kv1.3 blockade [13, 28]. Therefore, we characterized the phagocytic capacity of acutely isolated CD11b^+^ CNS-MPs following tMCAO at various timepoints using a validated flow cytometric assay [16]. We observed that microsphere phagocytosis by CD11b^+^ CNS-MPs peaked at 48 h post-tMCAO in both ipsilateral (*p =* 0.008) and contralateral (*p =* 0.03) hemispheres although this augmentation was more pronounced in the ipsilateral hemisphere (Fig. 5). Among CNS-MP subsets, phagocytic augmentation was observed at 48 h in both CD45^low^ and CD45^high^ CNS-MPs although CD11b^+^CD45^high^ cells were more avid phagocytosers at all timepoints. We also observed an augmentation of phagocytic activity, both ipsilaterally and contralaterally (Fig. 5c). There was no correlation between phagocytic activity and Kv1.3 channel expression in our study (Additional file 5: Figure S5). These results suggest that CNS-MPs globally augment phagocytic activity in the ischemic brain and CNS-MP phagocytic activity in the tMCAO model does not appear to be coupled to Kv1.3 channel expression.

## Discussion

Microglial activation and CNS infiltration by peripheral myeloid cells following ischemic stroke can impact infarct expansion, edema, and neuronal survival [31]. Disease-modifying roles for CNS-MPs in ischemic stroke are suggested by microglia depletion studies resulting in exacerbation of neuroinflammation and brain injury in focal ischemic stroke models [32]. In neurodegeneration, microglia progressively transition from homeostatic to DAM states [12, 33, 34] and within DAM, we have identified distinct pro-inflammatory and anti-inflammatory DAM sub-profiles with potentially opposing functional roles [9, 13]. Although the relevance of the homeostatic and DAM paradigm to ischemic stroke is unclear, anti-inflammatory CNS-MP phenotypes have been observed in the first 1–2 days after which pro-inflammatory phenotypes predominate in rodent MCAO models [7]. Therefore, selective neuro-immunomodulatory therapies for stroke are needed that inhibit pro-inflammatory CNS-MP responses and shift CNS-MPs towards protective anti-inflammatory and homeostatic states rather than non-specific anti-microglial strategies [6, 32, 35, 36].

The Kv1.3 potassium channel has been identified as a promising therapeutic target to inhibit pro-inflammatory CNS-MP responses in neurodegenerative disease [15, 24, 28] as well as ischemic stroke models [14], resulting in improved neuropathological outcomes. Kv1.3 is highly co-expressed with pro-inflammatory DAM genes (Il1b, Tlr2, Hif1a, Ptgs2) and is a key regulator of pro-inflammatory CNS-MP responses [13, 28, 29, 37]. Kv1.3 channels regulate membrane potential, calcium flux, immune signaling, and effector functions of effector memory T cells, memory B cells, and subsets of activated microglia and macrophages [38, 39]. Neuronal Kv1.3 expression is limited to olfactory and cortical neurons as hetero-tetramers with other Kv1-family channels while immune cells exclusively express the homo-tetrameric channel [38, 39]. This pattern of expression allows selective blockade of immune Kv1.3 channels by highly selective small molecule blockers (Pap1) and sea anemone toxin-based peptides (ShK analogs) without undesired off-target effects [40, 41]. An early phase study of Kv1.3 channel-blocking ShK analog (ShK-186/dalazatide) in humans has demonstrated safety, supported by multiple pre-clinical studies showing safety and efficacy of Kv1.3 blockers in models of systemic autoimmunity, obesity, CNS demyelinating disorders, and neurodegeneration [15, 18, 28, 42–44], suggesting that rapid translation of Kv1.3 blockers to ischemic stroke is feasible [45]. To further build on the pre-clinical rationale of Kv1.3 channel blockade as a therapeutic approach in ischemic stroke, the key CNS immune cells targeted by Kv1.3 blockers and the optimal therapeutic window for Kv1.3 blockers need to be determined.

We have utilized a rapid flow cytometric assay of functional cell-surface Kv1.3 channels [13, 18, 21] to phenotype acutely isolated CNS-MPs following tMCAO, and found that functional Kv1.3 channel expression is increased specifically in CD11b^+^CD45^low^Ly6c^low^ resident microglia as well as in the subset of CD11b^+^CD45^high^Ly6c^low^ CNS-MPs, but not in CD11b^+^CD45^high^Ly6c^high^ inflammatory monocytes. Therefore, it is highly likely that microglia are the primary cell types targeted by Kv1.3 blockers and that the beneficial effects of Kv1.3 blockers in stroke models  are unlikely to be mediated via modulation of peripheral immune responses. We also found increased Kv1.3 expression in the CD11b^+^CD45^high^Ly6c^low^ population of CNS-MPs which may represent an activated population of microglia or non-inflammatory patrolling monocytes that are recruited to the brain [26]. We also observed that not all microglia increase Kv1.3 expression, suggesting that additional immune sub-profiling of microglia may provide novel biological insights into the regulation and roles of Kv1.3 channel expression in microglial activation [46]. Increased functional cell-surface Kv1.3 channel expression at 48 h post-tMCAO was not accounted for by mRNA-level changes in Kv1.3, implicating post-transcriptional and post-translational processes in channel regulation in microglia [46]. However, this interpretation is limited by the large variance in our qRT-PCR data, insufficient mRNA yields from CNS-MP subsets and the use of all CNS-MPs (which contain 60% CD11b^+^ CNS-MPs), rather than enriched CD11b^+^ CNS-MPs within the scope of this study. Based on increased Kv1.3 channel expression between 24 and 72 h followed by a decrease by 7 days post-tMCAO, we also define the therapeutic window for Kv1.3 blockers as the subacute/delayed phase of ischemic stroke rather than hyper-acute/acute timeframes.

Our tMCAO studies were limited to 30 min of vessel occlusion followed by reperfusion. This model was specifically selected to minimize mortality observed with longer durations of occlusion especially beyond 48 h post-tMCAO. Although we did not characterize Kv1.3 channel expression by flow cytometry in longer MCAO models, other groups have shown in the 60-min MCAO model that Kv1.3 protein expression was indeed increased between day 2 and day 8 post-MCAO, suggesting that our findings regarding delayed Kv1.3 channel expression by microglia seems to be consistent regardless of the duration of vessel occlusion [14]. As compared to traditional electrophysiological methods such as a whole-cell patch clamp which has very limited sampling capabilities (< 40–50 cells per mouse), our flow cytometric approach to characterize Kv1.3 channel expression provides a rapid and comprehensive strategy which also allows us to assess Kv1.3 expression within CNS-MP subsets. Another interesting finding that we report is the downregulation of Tmem119 protein in microglia at 48 h post-tMCAO, which suggests that microglia downregulate homeostatic genes/proteins and may potentially transition towards activated phenotypes following MCAO [11–13, 28]. Whether these delayed activated phenotypes share any similarities with DAM phenotypes observed in neurodegeneration remains to be explored. We also did not note any relationship between phagocytic activity in CNS-MPs and Kv1.3 channel expression. The overall increase in phagocytic activity in both hemispheres at 48 h post-tMCAO may indicate a global response although the mechanisms or implications of this observation remain unexplored. Our results with phagocytic uptake of latex microspheres also do not necessarily reflect the ability of CNS-MPs to clear cellular debris in the ischemic core or to phagocytose healthy neuronal synapses as these processes involve specific receptors including complement receptors [3, 47]. Ongoing studies investigating specific mechanisms of phagocytosis in post-stroke CNS-MPs, as well as transcriptomic and proteomic efforts to characterize CNS-MPs in stroke models, will provide additional clarification.

From a clinical perspective, our findings in the transient 30-min MCAO model are relevant to acute large-vessel occlusion (LVO) stroke, which represent 25–40% of acute ischemic stroke [1, 48]. With the advent of mechanical thrombectomy as a highly effective reperfusion strategy, acute LVO stroke patients can be treated up to 24 h post-onset of stroke symptoms. However, nearly 50% of patients still suffer significant disability [9] and very few effective therapies exist beyond the 24-h window [10, 49]. Since reperfusion can be established in LVO patients, the stroke field is ideally poised to revisit neuroprotective and neuro-immunomodulatory strategies such as Kv1.3 blockade to target disease mechanisms most relevant in the post-acute phases of ischemic stroke.

## Conclusions

We have shown that Kv1.3 channels are specifically and highly expressed by resident microglia and a subset of CD45^high^Ly6c^low^ CNS-MPs and are the likely cellular targets of Kv1.3 blockers. Furthermore, we suggest that the delayed phase of neuroinflammation is the optimal therapeutic window for Kv1.3 blockade in ischemic stroke.

## Additional files

Additional file 1: **Figure S1.** Confirmation of effective tMCAO and reperfusion in mice. (DOCX 503 kb)

Additional file 2: **Figure S2.** Alterations in proportions of CD45high CNS-MP subsets following tMCAO. (DOCX 319 kb)

Additional file 3: **Figure S3.** Minimal cell-surface Kv1.3 expression by splenic monocytes. (DOCX 475 kb)

Additional file 4: **Figure S4.** Immunohistochemical validation of Kv1.3 protein expression by microglia following tMCAO (related to Fig. 3). (DOCX 3630 kb)

Additional file 5: **Figure S5.** Correlation between functional Kv1.3 channel expression and phagocytic capacity for fluorescent microspheres in acutely isolated CNS-MPs following tMCAO. (DOCX 26 kb)

## Data Availability

All data generated or analyzed during this study are included in this published article.

## Abbreviations

CNS-MPs: Central nervous system mononuclear phagocytes
DAM: Disease-associated microglia
Kv1.3: Voltage-gated potassium channel 1.3
tMCAO: Transient middle cerebral artery occlusion
