# Supplementary material for: Temporal profiling of Kv1.3 channel expression in brain mononuclear phagocytes following ischemic stroke
Source: J Neuroinflammation. 2019 Jun 1;16:116. doi: 10.1186/s12974-019-1510-8 (PMC6545199; doi:10.1186/s12974-019-1510-8)
Supplement: Supplementary file 1 — Figure S1. Confirmation of effective tMCAO and reperfusion in mice. (DOCX 503 kb) [file 12974_2019_1510_MOESM1_ESM.docx]

**
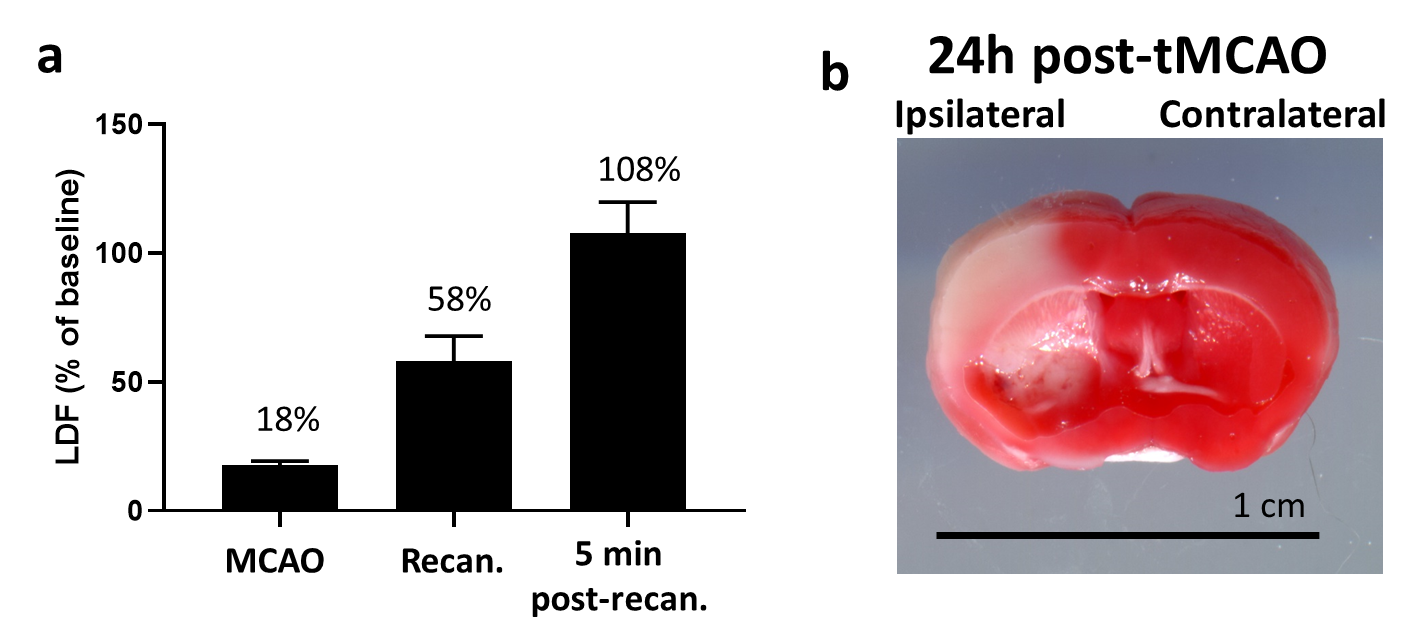
Additional file 1: Figure S1. Confirmation of effective tMCAO and reperfusion in mice.** (a) Relative laser doppler flow (LDF) data at time of MCAO, at time of recanalization as well as at 5 min post-recanalization (normalized to baseline LDF). LDF data from mice included in the study have been summarized above in (a). Error bars represent standard error of mean (SEM). (b) Representative TTC stained coronal section of post-tMCAO brain at the 24h timepoint. Infarct is clearly visualized in the ipsilateral hemisphere, confirming successful MCAO.
