# Supplementary material for: Temporal profiling of Kv1.3 channel expression in brain mononuclear phagocytes following ischemic stroke
Source: J Neuroinflammation. 2019 Jun 1;16:116. doi: 10.1186/s12974-019-1510-8 (PMC6545199; doi:10.1186/s12974-019-1510-8)
Supplement: Supplementary file 2 — Figure S2. Alterations in proportions of CD45high CNS-MP subsets following tMCAO. (DOCX 319 kb) [file 12974_2019_1510_MOESM2_ESM.docx]

**
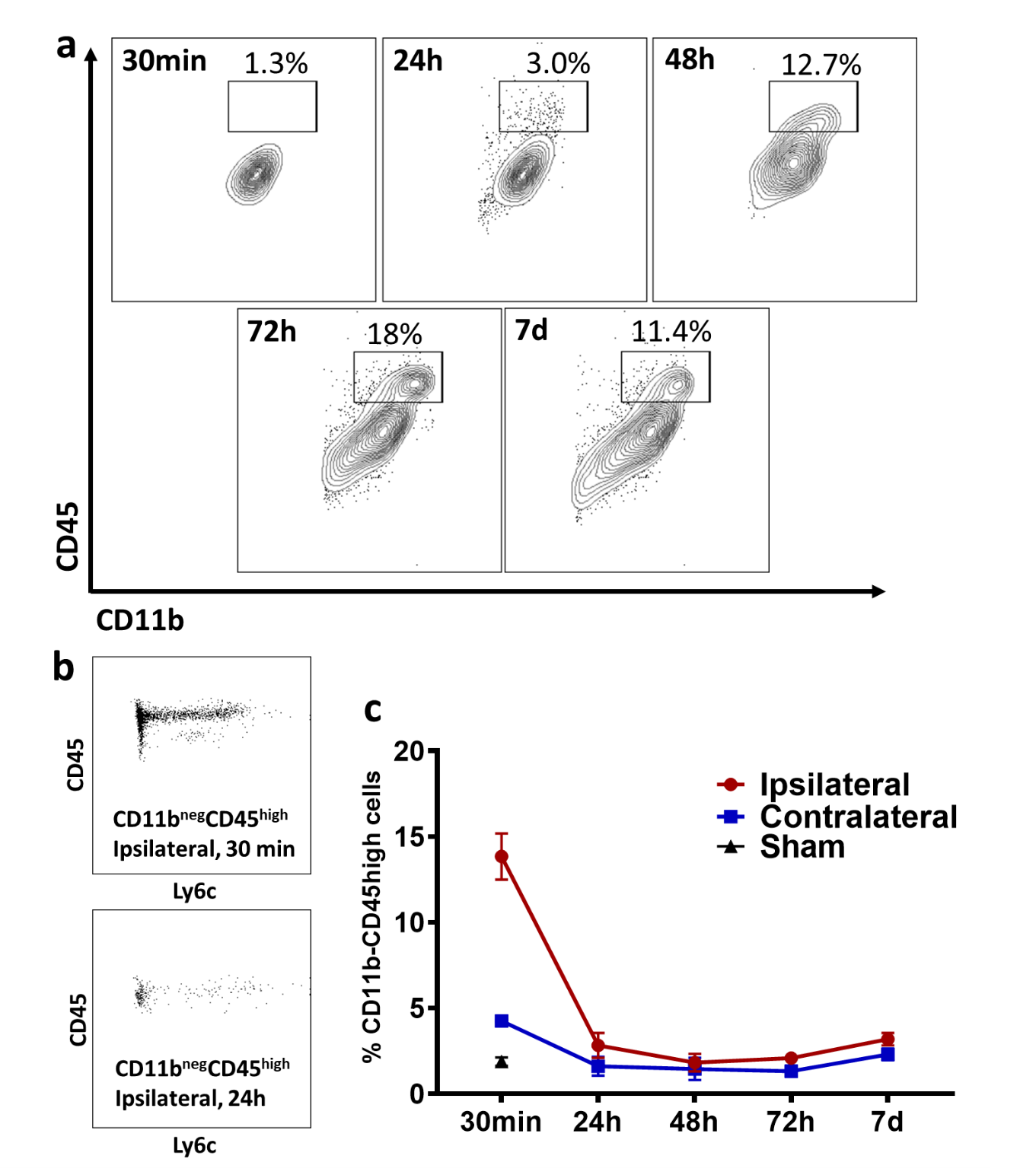
**

**Additional file 2: Figure S2. Alterations in proportions of CD45^high^ CNS-MP subsets following tMCAO.** (a) Proportion of CD45^high^ CNS-MPs within CD11b^+^ CNS-MPs at various timepoints post-tMCAO (related to Figure 1b). (b) Flow cytometric scatter plots of CD11b^neg^ CD45^high^ cells (lymphocytes) isolated from the ipsilateral hemispheres of mice at 30 min (top) and 24h (bottom) post-tMCAO. (c) Comparison of proportions of CD11b^neg^ CD45^high^ cells at various timepoints post-tMCAO in ipsilateral and contralateral hemispheres.
