# Supplementary material for: Temporal profiling of Kv1.3 channel expression in brain mononuclear phagocytes following ischemic stroke
Source: J Neuroinflammation. 2019 Jun 1;16:116. doi: 10.1186/s12974-019-1510-8 (PMC6545199; doi:10.1186/s12974-019-1510-8)
Supplement: Supplementary file 3 — Figure S3. Minimal cell-surface Kv1.3 expression by splenic monocytes. (DOCX 475 kb) [file 12974_2019_1510_MOESM3_ESM.docx]

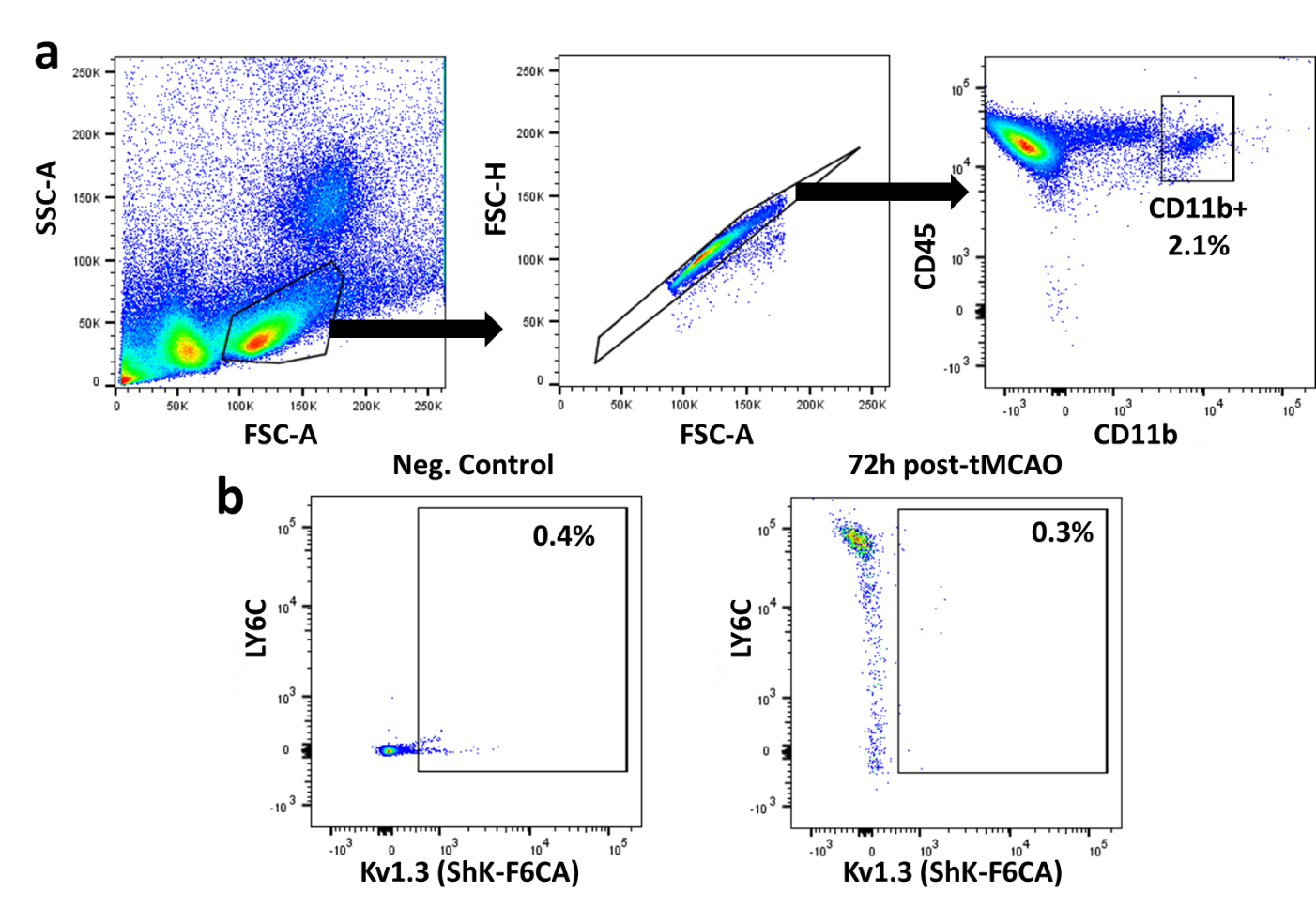
**Additional file 3: Figure S3. Minimal cell-surface Kv1.3 expression by splenic monocytes**. (a) Gating strategy for splenocytes: Gate 1 selects for live mononuclear cells excluding neutrophils. Gate 2 selects single live cells. Gate 3 identifies CD11b+ myeloid cells. CD11b+ cells were then evaluated for Ly6c and Kv1.3 expression. (b) As compared to negative control (left), Ly6c high, Ly6c intermediate and Ly6c low monocytes had almost no detectable surface Kv1.3 channel expression following transient MCAO. Example of one 72h post-tMCAO spleen is shown.
