# Supplementary material for: Temporal profiling of Kv1.3 channel expression in brain mononuclear phagocytes following ischemic stroke
Source: J Neuroinflammation. 2019 Jun 1;16:116. doi: 10.1186/s12974-019-1510-8 (PMC6545199; doi:10.1186/s12974-019-1510-8)
Supplement: Supplementary file 4 — Figure S4. Immunohistochemical validation of Kv1.3 protein expression by microglia following tMCAO (related to Fig. 3). (DOCX 3630 kb) [file 12974_2019_1510_MOESM4_ESM.docx]

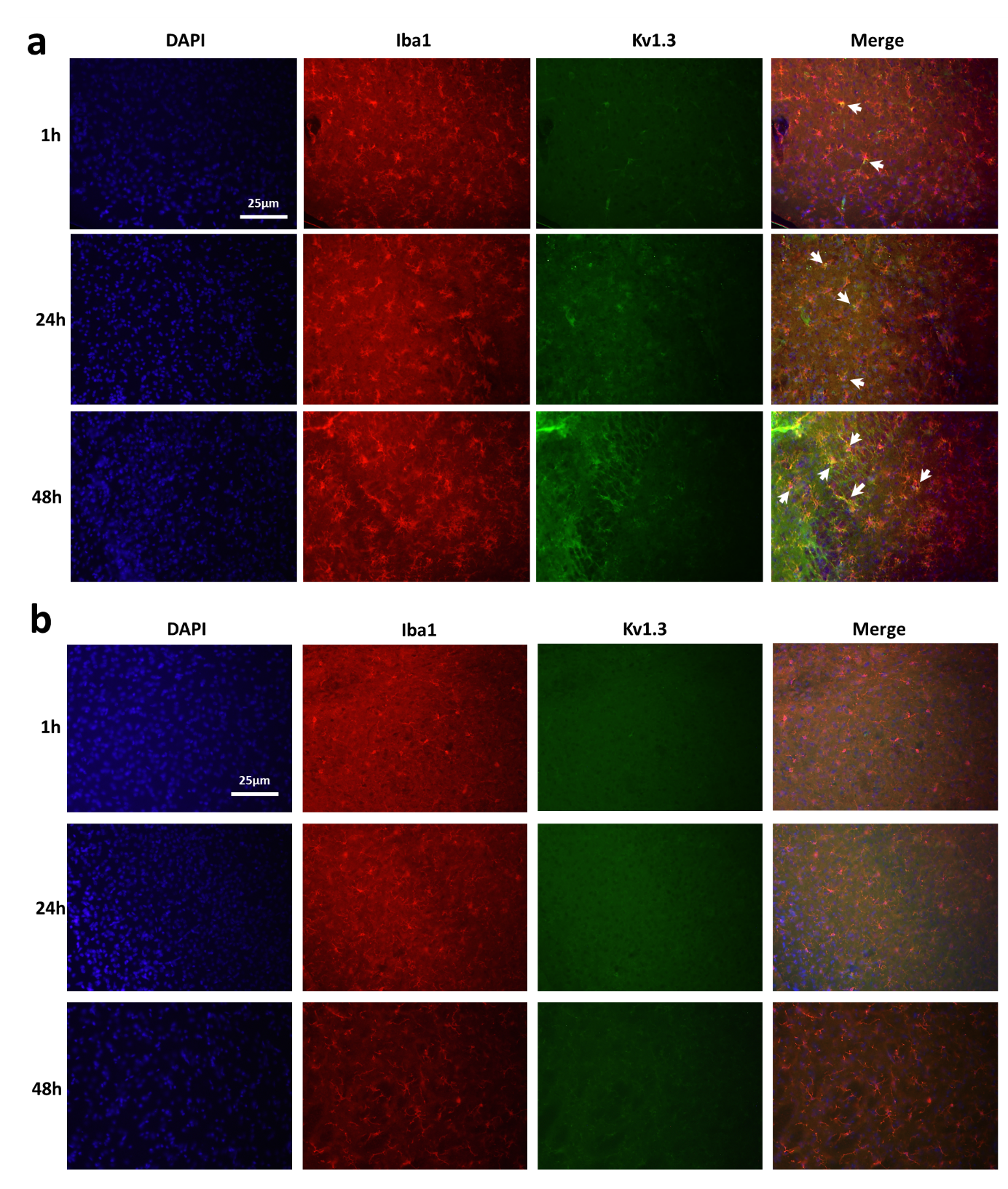
**Additional file 4: Figure S4. Immunohistochemical validation of Kv1.3 protein expression by microglia following tMCAO (related to Figure 3).** Immunofluorescence micrographs from brain sections showing microglia marker Iba1 and Kv1.3 channel expression at 1h, 24h and 48h post-tMCAO. (a) In the ipsilateral hemispheres, Iba1+ CNS-MPs (Red) shows gradual increase in kv1.3 expression (Green) at 24h and 48h as compared to 1hr post-tMCAO. The white arrows highlight areas of co-localization between Kv1.3 and Iba1. (b) In the contralateral hemispheres, low Kv1.3 (Green) immunoreactivity was observed in Iba1^+^ CNS-MPs (Red).
