# Supplementary material for: Temporal profiling of Kv1.3 channel expression in brain mononuclear phagocytes following ischemic stroke
Source: J Neuroinflammation. 2019 Jun 1;16:116. doi: 10.1186/s12974-019-1510-8 (PMC6545199; doi:10.1186/s12974-019-1510-8)

**Additional file 5: Figure S5.** **Correlation between functional Kv1.3 channel expression and phagocytic capacity for fluorescent microspheres in acutely-isolated CNS MPs following tMCAO**. Samples collected from ipsilateral and contralateral hemispheres were treated as independent events and PE-microsphere phagocytosis and functional Kv1.3 channel measurement by ShK-F6CA were performed in parallel on the sample samples.
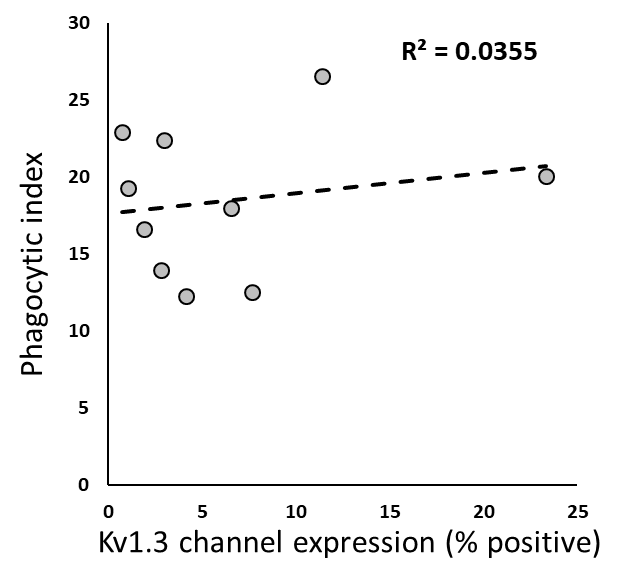

Supplement: Supplementary file 5 — Figure S5. Correlation between functional Kv1.3 channel expression and phagocytic capacity for fluorescent microspheres in acutely isolated CNS-MPs following tMCAO. (DOCX 26 kb) [file 12974_2019_1510_MOESM5_ESM.docx]
